# Effect of Electric Field on α-Synuclein Fibrils: Revealed by Molecular Dynamics Simulations

**DOI:** 10.3390/ijms24076312

**Published:** 2023-03-28

**Authors:** Jamoliddin Razzokov, Sunnatullo Fazliev, Mukhriddin Makhkamov, Parthiban Marimuthu, Artyom Baev, Erkin Kurganov

**Affiliations:** 1 Institute of Fundamental and Applied Research, National Research University TIIAME, Kori Niyoziy 39, Tashkent 100000, Uzbekistan; 2 R&D Center, New Uzbekistan University, Mustaqillik Avenue 54, Tashkent 100007, Uzbekistan; 3 Institute of Material Sciences, Academy of Sciences, Chingiz Aytmatov 2b, Tashkent 100084, Uzbekistan; 4 Department of Physics, National University of Uzbekistan, Universitet 4, Tashkent 100174, Uzbekistan; 5 Max Planck School Matter to Life, Jahnstrasse 29, 69120 Heidelberg, Germany; 6 Faculty of Engineering Sciences, Heidelberg University, Im Neuenheimer Feld 205, 69120 Heidelberg, Germany; 7 Laboratory of Experimental Biophysics, Centre for Advanced Technologies, Tashkent 100174, Uzbekistan; 8 Pharmaceutical Science Laboratory (PSL–Pharmacy) and Structural Bioinformatics Laboratory (SBL–Biochemistry), Faculty of Science and Engineering, Åbo Akademi University, FI-20520 Turku, Finland; 9 Department of Biophysics, Biological Faculty, National University of Uzbekistan, Universitet 4, Tashkent 100174, Uzbekistan; 10 Stanley Center for Psychiatric Research, Broad Institute of MIT and Harvard, Cambridge, MA 02142, USA

**Keywords:** Parkinson’s disease, α-synuclein, molecular dynamics, electric field, fibril disaggregation

## Abstract

The self-association of amylogenic proteins to the fibril form is considered a pivotal factor in the pathogenesis of neurodegenerative diseases, including Parkinson’s disease (PD). PD causes unintended or uncontrollable movements in its common symptoms. α-synuclein is the major cause of PD development and thus has been the main target of numerous studies to suppress and sequester its expression or effectively degrade it. Nonetheless, to date, there are no efficient and proven ways to prevent pathological protein aggregation. Recent investigations proposed applying an external electric field to interrupt the fibrils. This method is a non-invasive approach that has a certain benefit over others. We performed molecular dynamics (MD) simulations by applying an electric field on highly toxic fibrils of α-synuclein to gain a molecular-level insight into fibril disruption mechanisms. The results revealed that the applied external electric field induces substantial changes in the conformation of the α-synuclein fibrils. Furthermore, we show the threshold value for electric field strength required to completely disrupt the α-synuclein fibrils by opening the hydrophobic core of the fibril. Thus, our findings might serve as a valuable foundation to better understand molecular-level mechanisms of the α-synuclein fibrils disaggregation process under an applied external electric field.

## 1. Introduction

Neurodegenerative diseases, a group of late-onset progressive nervous system diseases, posed a severe challenge before modern medicine. Parkinson’s disease (PD) is a widespread neurological disorder that is pathologically characterized by progressive loss of dopaminergic neurons [1,2]. Aside from common motor disorders such as bradykinesia, tremor, rigidity, and postural instability [3], PD also severely affects the quality of life through complications such as cognitive impairment, mental health disorders, sleep disorders, and pain [4]. The hypothesis that protein aggregation might be a cause of neurodegenerative diseases, including PD, is now being widely acknowledged as evidenced by numerous medical [5], biochemical [6,7], and biophysical [8] studies that demonstrated that neurodegenerative diseases are not only caused by diverse environmental factors, but genetic factors play a crucial role too [3,9,10].

Several neurodegenerative diseases, such as PD, dementia with Lewy bodies, and Gaucher’s disease, are caused by α-synuclein through protein aggregation pathways and can be spread to other parts of the nervous system [11,12,13,14]. α-synuclein is a small protein (M_w_ = 14 kDa) with an intrinsically disordered structure. The physiological functions of monomeric α-synuclein are debatable [15], though there are some reports that show its involvement in vesicular transport [16], cellular bioenergetics [17], and immunity [18]. Its aggregated form is the main component of filamentous inclusions known as Lewy bodies—a defining pathological characteristic of PD [19]. Familial PD-related point mutations (A30P, E46K, H50Q, G51D, A53E, and A53T) occur in a 60-amino-acid-long, lysine-rich N-terminal region of α-synuclein [20]. The rest of this 140-amino-acid-long protein is of hydrophobic nature: the 35-amino-acid-long residue (61–95) comprises a non-amyloid β-component region and is followed by a proline-rich C-terminal region (amino acids 96–140) [21].

As with other proteins prone to aggregation, α-synuclein also seems to be neglected by the protein quality control system, whose proper work should prevent aberrant protein structures [22]. At a physiological pH, α-synuclein possesses a net negative charge and behaves like an unfolded polypeptide chain (70% disordered structure) with the radius of gyration (R_g_) being only 4 nm (more compact than a typical random coil of 140 amino acids long) [9]. α-synuclein’s remarkable structural plasticity allows it to exist largely unfolded at physiological conditions, to fold at low pH and high temperatures, or to aggregate when exposed to environmental changes such as acidity, oxidative damage, and metal ions. Such changes are very plausible in the cellular environment. In addition, it has been shown that α-synuclein aggregates are able to self-propagate different conformational variants (also called “conformational strains”) which, in turn, can produce fibrils of different properties resulting in their polymorphic nature [23,24]. Hence, besides genetic modifications, certain environmental factors contribute to the conformational changes of α-synuclein that facilitate aggregation and fibril formation. Therefore, α-synuclein, as a hallmark of PD pathogenesis, is the main target of PD diagnosis and treatment. This is evident by numerous contemporary attempts where PD treatment is sought through small molecule drugs, gene therapy, and immunotherapy approaches to sequester, silence, and degrade α-synuclein [13,25,26,27].

Changes in polarity and electric and/or magnetic fields also affect the structure and functions of biomolecules that possess considerably larger dipoles than small molecules. In this modern era of technology, we are increasingly exposed to electromagnetic radiation through various gadgets. Several studies have been conducted to study the effects of externally applied electric field (EF) at the molecular, cell, and tissue levels [28]. The electric field, as an external stimulus is also of great interest for neurodegenerative disease research, too. For instance, for 25 years, deep brain stimulation (DBS) through an oscillating electric field has been used to treat and improve the quality of life in late-stage PD patients [29]. Recently, the focus of neurodegenerative disease research has been shifted towards the molecular level: on the effects of electric field on protein conformation, aggregation, and fibril formation. It has already been shown that different electric field modalities, such as oscillating [30,31] and static fields [32,33] can induce significant structural changes in amylogenic proteins [34,35] and disrupt aggregations and fibrils [36]. Studies also report electric field-induced changes in activity [37,38], hydration [39], and adsorption [40] of proteins. Molecular dynamics (MD) simulation is one of the leading tools to study biomolecular phenomena due to the valuable molecular information that can be obtained. Recent years have seen many MD studies dedicated to investigating the external electric field’s influence on protein conformation [41,42,43]. MD simulations offer atomistic-level insights into individual amylogenic protein conformations and their aggregates. For instance, the full-length α-synuclein monomer and dimer were investigated by means of atomistic discrete MD simulations [44]. The modeling results predicted the formation of partial helices around the N-terminus (residues 8–32). The different types of β-sheet conformation occurred in the range of residues 35–56 (N-terminal tail) and residues 61–95 (nonamyloid β-component region). In α-synuclein dimers, some disordered parts of the α-synuclein conformationally transformed into the β-sheet conformation. Other simulation studies also show the importance of dimerization in triggering the α-synuclein aggregation by conformational transformations into both intramolecular β-hairpin and β-sheet [45]. Moreover, the effect of specific conditions, e.g., pH and ions and charge alterations, were also studied by applying specific computational methods [46,47,48]. Thus, knowledge about the nature of interactions between certain regions of α-synuclein plays a critical role in preventing its aggregation [49]. Such precise information and biochemistry findings will help us develop a mechanistic understanding of protein aggregation diseases and ultimately triumph over such disorders.

In the present research, we use MD simulations to study electric field-induced changes in α- synuclein fibril conformation. In addition, we unravel the threshold value of the electric field for total disaggregation of α-synuclein fibrils.

## 2. Results and Discussion

**Figure 1:**
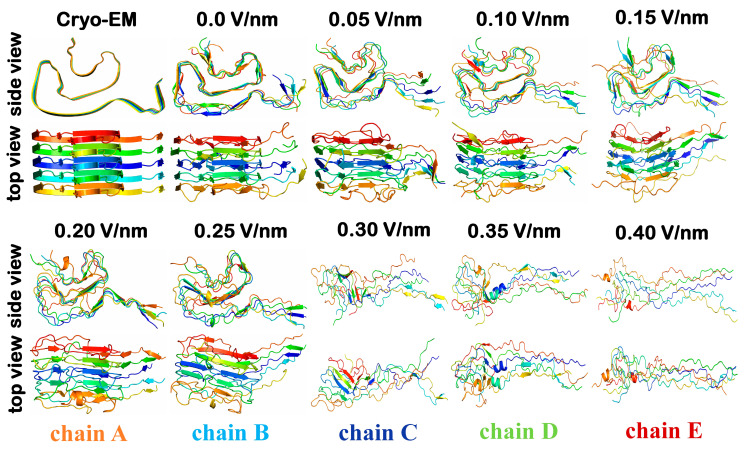
The last snapshots of α-synuclein pentamers which were extracted from the 600 ns MD trajectory. The side and top view show conformational changes on α-synuclein under the influence of 0.05, 0.10, 0.15, 0.20, 0.25, 0.30, 035, and 0.40 V/nm electric field.

We carried out MD simulations to investigate the static EF effect on the conformational changes of the α-synuclein fibril. The chosen mutated α-synuclein H50Q narrow fibril structure displays a tendency for faster aggregation kinetics and higher toxicity in comparison to the wild type α-synuclein structure [50]. Thus, the disruption of such fibrils is important in combat against amyloid-based diseases, including PD. Figure 1 shows the final snapshots of a replica 1 (out of four) of the 600 ns MD simulation of the α-synuclein pentamer structure.

As is clear, there is almost no change in the α-synuclein pentamer in the absence of EF, i.e., its conformation is quite similar to experimental findings. The low values of EF, e.g., 0.05 and 0.10 V/nm, induced negligible change. However, the N-terminal end of the α-synuclein pentamer (i.e., residues between 36–46) unfolded and moved further from the main core of fibril, starting from 0.05 V/nm EF. Similar alterations in conformation were observed in the case of 0.15, 0.20 and 0.25 V/nm EF. Moreover, the secondary structure analysis shows a 17% reduction of the β-sheet conformation for 0.25 V/nm (see Table 1). Note that the β-sheet conformation plays one of the dominant roles in stabilizing fibril-like structures [51]. The further increase in the EF strength from 0.30 to 0.40 V/nm, caused even more impact on fibril conformation. Specifically, β-sheet conformation decreased almost four times and the major percentage of this conformation transformed to the coil conformation, which was doubled in 0.4 V/nm in comparison to the absence of EF (cf. β-sheet and coil conformation in Table 1). Moreover, the helical conformation also started emerging in higher intensity of EF (see Supplementary information Figure S1). Thus, the low intensity of EF such as 0.05–0.25 V/nm was sufficient to induce conformational changes but the core of the fibril remained. In contrast, the higher values of EF, e.g., 0.3–0.4 V/nm, caused more changes that resulted in the opening of fibril’s core and mainly turned it to coil conformation (cf. Figure 1, Figure S1, and Table 1).

**Figure 2:**
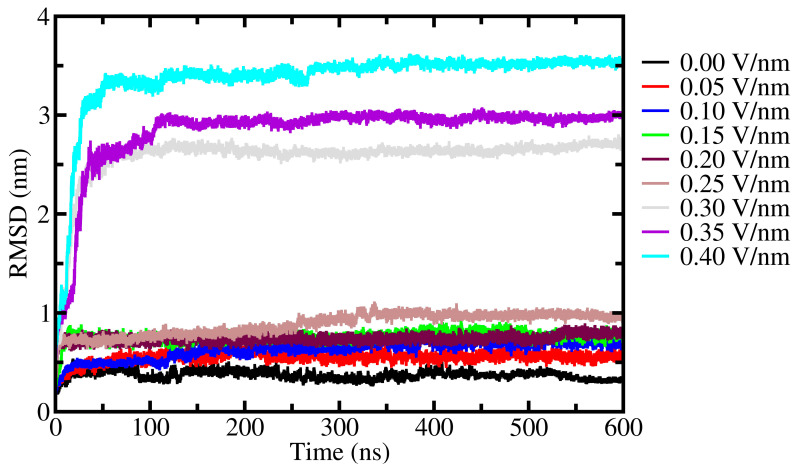
The RMSD of backbone structure of α-synuclein fibril.

The calculated backbone root mean square deviation (RMSD) plot shows that 0.05–0.25 V/nm EF strength disturbed fibril structure and caused higher fluctuations compared to the absence of the EF (see Figure 2). However, these fluctuations lead only to the unfolding of the N-terminal end, i.e., residues from 36 to 46, and the hydrophobic core remained unchanged (see Figure 1). However, 0.30–0.40 V/nm EF induced more changes in the conformation of the α-synuclein structure, in that the occurrence of β-sheet conformation decreased significantly, and this conformation mainly was altered into coil conformations (see Table 1 and Figure S1). Finally, in the case of 0.40 V/nm EF, the α-synuclein fibril completely unfolded (see Figure 1) and the hydrophobic core of the α-synuclein fibril completely opened which was remained at lower EF strengths. According to the RMSD, the major change occurs within the initial 100 ns simulation, i.e., in high EF intensity such as 0.3, 0.35, and 0.4 V/nm (see Figure 1). The secondary structure map also shows insignificant changes in conformation in the rest of the simulation time (see Figure S1).

The RMSD values of backbone atoms increased almost ten times as the EF strength increased (see Figure 2). Likewise, the solvent accessible surface area (SASA) and radius of gyration (R_G_) of fibril increased at higher EF strengths. The SASA and R_g_ of fibrils reached the highest values and the conformation of each peptide became almost linear, similar to the primary structure of proteins. Furthermore, under this condition, the narrow and uniform shape of the α-synuclein fiber turned into a flat form which marked a full disaggregation point for the α-synuclein fiber. In addition, the root mean square fluctuations (RMSF) was calculated to understand the flexibility and dynamics of different regions of the peptide located in the middle of the pentamer chain C (see Figure S2). The aim of choosing the latter is associated with its stability and this chain is highly buried by neighboring chains. It is also evident that the presence of EF influenced the mobility of residues. As a result, fluctuations of chain C had considerably changed and showed greater values between 60 and 90 residues at 0.3 and 0.4 V/nm EF intensity (see Figure S2).

Interprotein interactions, specifically in fiber-like proteins, lateral hydrogen bonds between peptides, hydrophobic packing of residues, and salt bridges play a vital role in stabilizing and stimulating further elongation of fibrils [52,53,54]. The extensive number of hydrogen bonds between individual β-strands and long-range interactions drive cytotoxic fibril formation [55]. Our results show that the average number of the inter- and intrapeptide hydrogen bonds per chain gradually decreased under the influence of EF (see Table 2). This hydrogen bond loss affects α-synuclein fiber stability by lowering the strength of intrapeptide interactions. Thus, highly ordered β-sheet-rich fibrils are quite sensitive to EF.

**Figure 3:**
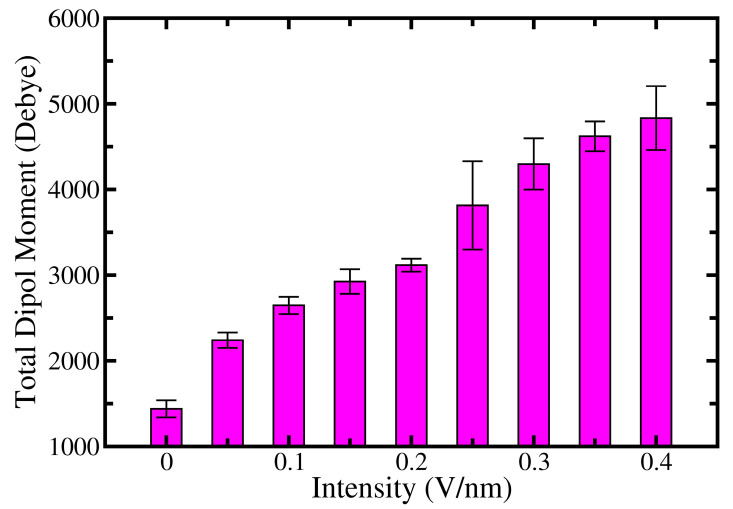
The total dipole moment of fibrillar complex depending on EF perturbation.

It is worth mentioning that each type of protein maintains a certain value of a dipole moment due to the presence of charged side chains [56,57]. The value of a total dipole moment can serve as one of the indicators that show conformational changes on the protein, i.e., its folded or denaturated state. An increase in total dipole moments in comparison to the native state of the protein is associated with conformational transitions towards to the denaturation state. Therefore, we also calculated the total dipole moments of the pentamer for each case of applied EF and the average over the all replicas (see Figure 3).

As is clear from Figure 3, in the absence of EF the total dipole moment of α-synuclein fiber is ~1400 Debye. Evidently, the presence of EF induces a force that acts on charged side chains. Consequently, the total dipole moment increased continuously and reached the highest value, i.e., ~4800 Debye, at 0.4 V/nm. In other words, the total dipole moment of the α-synuclein fiber rose more than three times compared to that in the absence of EF. Furthermore, we observed the fast change of orientation of α-synuclein fibril at higher values of applied EF. This in turn led the alignment of total dipole moments of α-synuclein fibril along the EF direction in a short period of time during the simulation (see Figure S3). The contribution of salt bridges is also substantial in holding the conformation of fibers [58,59,60]. In the current conformation, Glu46 and Lys80 form inter- and intrapeptide salt bridges, which prevent the opening of the fibril’s hydrophobic core at lower values of EF. 

## 3. Methods and Materials

The graphical processing unit (GPU) version of the GROMACS program package was employed to perform all simulations [61]. The united atom GROMOS 45a3 force field parameters were used to generate the necessary files to run the model system [62]. The 3D coordinate structure of α-synuclein H50Q narrow fibril was obtained from the web page of the Protein Data Bank (PDB ID: 6PEO) [50].

**Figure 4:**
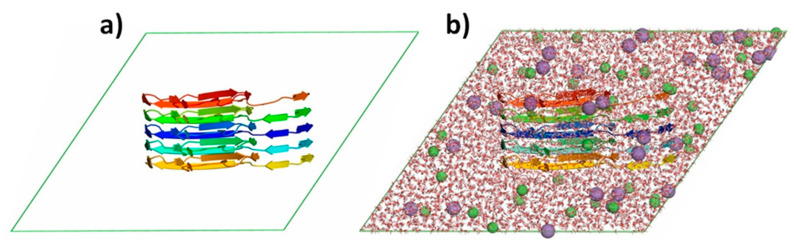
The representation of the initial state of the model system. (**a**) α-synuclein pentamer is placed in the center of the dodecahedron box (represented in cartoon view—rainbow color). (**b**) The simulation box filled with water molecules (represented in licorice view) and sodium and chloride ions (shown as violet and green beads).

In order to build the simulation system, the α-synuclein structure was centred in the dodecahedron box, and the dimensions of the current box were chosen to be 1.1 nm from atoms of α-synuclein to the edges of the box (see Figure 4a). Further, the system was filled by a simple point charge water model with 0.1 M NaCl to create a similar physiological environment (see Figure 4a,b) [63].

Initially, the energy minimization was run to remove the excess potential in the model system. During this simulation, atoms found the appropriate positions corresponding to the nearest local minimum energy conformation for the given model system. Next, short 100 ps NVT (canonical ensemble) and 500 ps NPT (isobaric-isothermal ensemble) simulations were performed by applying the position-restrained potential. Subsequently, a 600 ns four replica production run was performed by releasing position restrained potential and randomizing velocities by applying 0.05, 0.10, 0.15, 0.20, 0.25, 0.30, 0.35, and 0.40 V/nm static electric fields along the X direction. The velocity-rescaling thermostat [64] and the Parrinello–Rahman barostat [65] were applied at 310 K and 1 atm, respectively. A 1 nm cut-off radius was used in these simulations. The entire trajectory dataset was used to calculate the root mean square deviation (RMSD) [66], and the last 200 ns of trajectory data was employed to calculate the radius of gyration, solvent accessible surface area (SASA) [67], number of hydrogen bonds per chain, residual root mean square fluctuations (RMSF), and secondary structure analysis of α-synuclein. The DSSP tool was used to determine detailed conformational changes in the α-synuclein structure. Pymol and visual molecular dynamics viewer (VMD) software were used to create images [68,69]. Note that, the data and snapshots in the main text was obtained from replica 1 simulation. The rest of the data which belong to other replicas are given in supplementary material (see Figures S1–S4 and Table S1).

## 4. Conclusions

PD is a progressive movement disorder with other nonmotor symptoms. Because of our brain’s plasticity, PD symptoms appear only after more than 50–60% of dopaminergic neurons within the substantia nigra are already dead [70]. Electrical DBS of specific areas shows good results for PD. The associated drawback is that it loses its effectiveness over time. The main idea of DBS is to stimulate the electric activity of neurons, but its effect on a molecular level is not totally clear. In our research, we perform MD simulations to investigate the static EF effect on the conformational changes of the α-synuclein fibril. We showed that the application of 0.30, 0.35, and 0.4 V/nm EF during 600 ns disorganized α-synuclein fibrils. Typical DBS parameter settings of voltage, pulse width, and frequency range are from 1 to 3.5 V, 60 to 210 ms, and 130 to 185 Hz, respectively [71].

We believe that classical settings of DBS might be enough to disorganize α-synuclein fibrils in brain cells; however, it should be tested in further experiments.

The formation of the α-synuclein inclusions occurs by a generic process of misfolding, by which an ordinarily soluble protein converts into fibrillar aggregates via a series of oligomeric intermediates and, ultimately, the insoluble fibrils are deposited in the brain. Soluble oligomeric species generated during the formation of fibrils are the most neurotoxic species linked with the development of PD [72,73,74]. The kinetic of α-synuclein fibril formation can often be dominated by secondary nucleation events, such as fibril fragmentation, adding further elements of complexity to the kinetic process [75]. Disorganization and spread prevention of amyloid fibrils are some of the main goals for scientists involved in PD research. Disorganization of α-synuclein fibrils, which we saw during MD simulation, might possibly lead to the formation of toxic oligomeric structures, which might further undergo second nucleation events or structures that will be utilized by the protein quality control system. Thus, we assume that further research, both in silico and in vitro, is needed to understand whether the disruption of α-synuclein fibrils by EF has a positive or negative impact.

## Figures and Tables

**Table 1:** Secondary structure analysis of the α-synuclein pentamer in each model system. The various conformation occurrences (%) of protein’s various secondary structure components.

EF (V/nm)	α-Helix	3 10-Helix	β-Sheet	β-Bridge	Bend	Turn	Coil
0.0	0.0	0.0	49	2	11	6	32
0.05	0.0	0.0	40	3	11	7	38
0.10	0.0	1.0	40	2	14	5	39
0.15	0.0	1.0	40	2	11	6	39
0.20	1.0	1.0	38	2	12	5	40
0.25	0.0	1.0	32	2	13	7	44
0.30	0.0	1.0	19	6	20	4	49
0.35	4.0	0.0	20	2	15	4	54
0.40	3.0	1.0	13	5	10	3	64

**Table 2:** The solvent-accessible surface area, the radius of gyration and the number of hydrogen bonds per chain calculated from the last 200 ns of the simulation trajectory.

EF (V/nm)	SASA (nm^2^)	R Gyration (Å)	h-Bond/Chain
0.00	139.58 ± 2.38	2.013 ± 0.02	48.20 ± 1.75
0.05	140.52 ± 2.42	2.028 ± 0.01	47.08 ± 1.55
0.10	153.90 ± 2.65	2.129 ± 0.01	44.08 ± 1.47
0.15	153.27 ± 2.44	2.142 ± 0.01	45.63 ± 1.52
0.20	150.69 ± 2.75	2.136 ± 0.01	44.73 ± 1.50
0.25	152.02 ± 2.65	2.214 ± 0.01	45.68 ± 1.44
0.30	164.08 ± 4.01	3.150 ± 0.03	40.03 ± 1.65
0.35	166.54 ± 4.33	3.398 ± 0.02	40.30 ± 1.58
0.40	184.36 ± 4.32	4.179 ± 0.02	35.32 ± 1.83

## Data Availability

All the data is included in the main text.

## Supplementary Materials

The following supporting information can be downloaded at: https://www.mdpi.com/article/10.3390/ijms24076312/s1.
