# Supplementary material for: Effect of Electric Field on α-Synuclein Fibrils: Revealed by Molecular Dynamics Simulations
Source: Int J Mol Sci. 2023 Mar 28;24(7):6312. doi: 10.3390/ijms24076312 (PMC10094641; doi:10.3390/ijms24076312)
Supplement: Supplementary file 1 [file ijms-24-06312-s001.zip › ijms-2148173-supplementary.pdf]

International Journal of *Molecular Sciences*

MDPI

Article

Effect of Electric Field on α-Synuclein Fibrils: Revealed by Molecular Dynamics Simulations

Jamoliddin Razzokov [1,2,3,4,*,†], Sunnatullo Fazliev [5,6,†], Mukhriddin Makhkamov [7], Parthiban Marimuthu [8], Artyom Baev [7,9] and Erkin Kurganov [10]

1 Fundamental and Applied Research, National Research University TIIAME, Kori Niyoziy 39, Tashkent 100000, Uzbekistan
2 R&D Center, New Uzbekistan University, Mustaqillik Avenue 54, Tashkent 100007, Uzbekistan
3 Institute of Material Sciences, Academy of Sciences, Chingiz Aytmatov 2b, Tashkent 100084, Uzbekistan
4 Department of Physics, National University of Uzbekistan, Universitet 4, Tashkent 100174, Uzbekistan
5 Max Planck School Matter to Life, Jahnstrasse 29, 69120 Heidelberg, Germany
6 Faculty of Engineering Sciences, Heidelberg University, Im Neuenheimer Feld 205, 69120 Heidelberg, Germany
7 Laboratory of Experimental Biophysics, Centre for Advanced Technologies, Tashkent 100174, Uzbekistan
8 Pharmaceutical Science Laboratory (PSL–Pharmacy) and Structural Bioinformatics Laboratory (SBL–Biochemistry), Faculty of Science and Engineering, Åbo Akademi University, FI-20520 Turku, Finland
9 Department of Biophysics, Biological Faculty, National University of Uzbekistan, Universitet 4, Tashkent 100174, Uzbekistan
10 Stanley Center for Psychiatric Research, Broad Institute of MIT and Harvard, Cambridge, MA 02142, USA
* Correspondence: jamoliddin.razzokov@ifar.uz; Tel.: +998-90-116-23-20
† These authors contributed equally to this work.

Citation: Razzokov, J.; Fazliev, S.; Mukhriddin, M.; Marimuthu, P.; Baev, A.; Kurganov, E. Effect of Electric Field on α-Synuclein Fibrils: Revealed by Molecular Dynamics Simulations. *Int. J. Mol. Sci.* **2023**, *24*, 6312.https://doi.org/10.3390/ijms24076312

Academic Editor: Zhi Dong Zhou

Received: 23 December 2022
Revised: 24 February 2023
Accepted: 13 March 2023
Published: 28 March 2023

Copyright: © 2023 by the authors. Licensee MDPI, Basel, Switzerland. This article is an open access article distributed under the terms and conditions of the Creative Commons Attribution (CC BY) license (https://creativecommons.org/licenses/by/4.0/).

Supplementary material

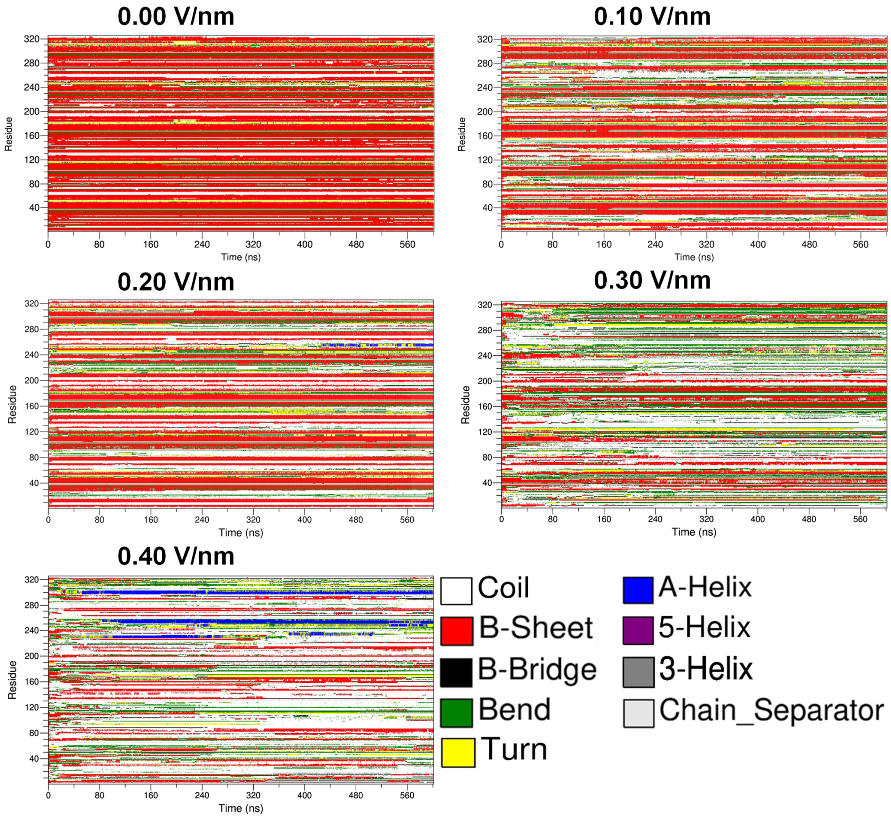

Figure S1. The secondary structure map of α-synuclein fibril under different EF intensities.

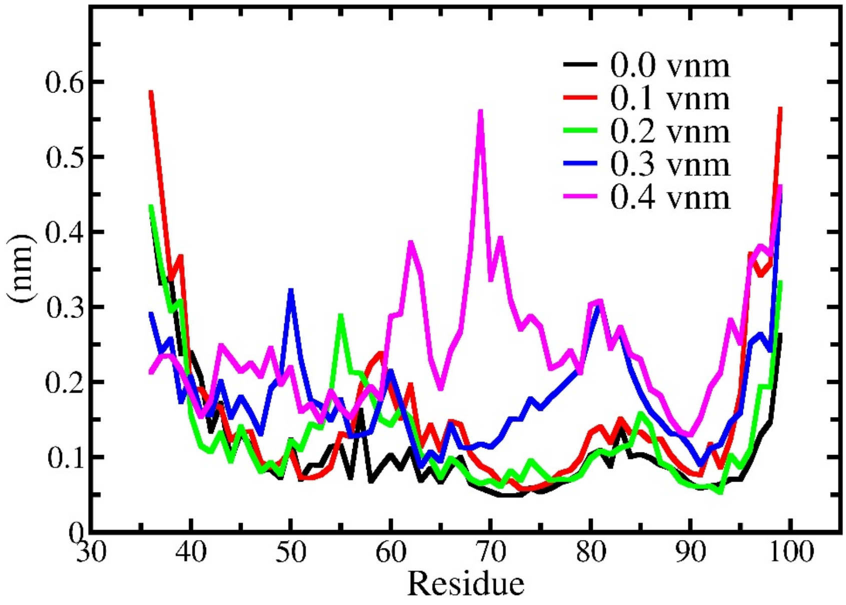

Figure S2. RMSF of chain C influenced by various EF strengths.

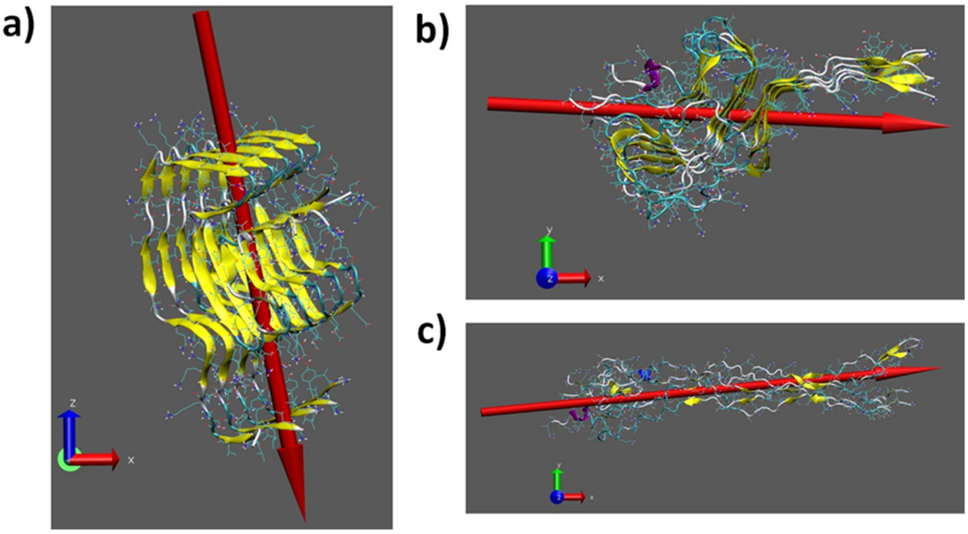

Figure S3. The representation of a total dipole moment of α-synuclein fibril in the case of (**a**) 0 V/nm, (**b**) 0.2 V/nm, and (**c**) 0.4 V/nm.

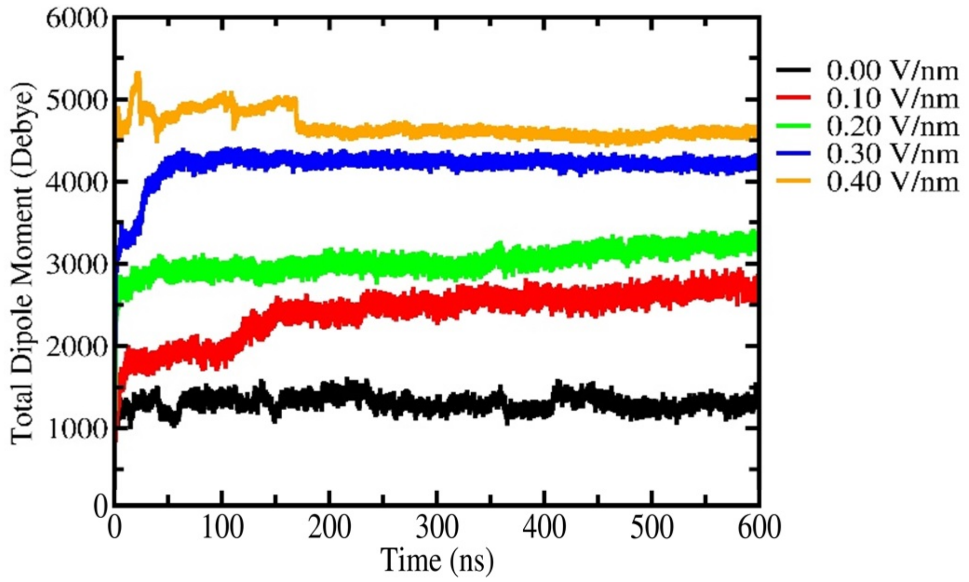

Figure S4. The time evolution of total dipole moment of α-synuclein fibril.

Table S1. The solvent-accessible surface area, the radius of gyration and the number of hydrogen bonds per chain calculated from the last 200 ns of the simulation trajectory for replica 1, 2, 3 and 4.

EF (V/nm)	Rep-lica	SASA (nm^2)	R.Gyration (Å)	h-bond/chain
0.10 M NaCl-neutral model system				
0.00	1	139.58±2.38	2.013±0.02	48.20±1.75
	2	137.95±3.40	2.022±2.66	47.11±2.23
	3	138.08±2.23	2.031±0.01	47.56±1.62
	4	141.36±2.37	2.046±0.01	45.87±2.08
0.05	1	140.52±2.42	2.028±0.01	47.08±1.55
	2	144.47±2.35	2.111±0.01	45.74±1.45
	3	142.73±2.60	2.092±0.02	46.73±1.57
	4	148.04±4.34	2.123±0.03	45.77±1.87
0.10	1	153.90±2.65	2.190±0.01	44,08±1.47
	2	147.47±2.78	2.090±0.01	46.77±1.63
	3	155.85±2.91	2.126±0.01	44.63±1.70
	4	153.41±3.05	2.099±0.01	45.11±1.52
0.15	1	153.27±2.44	2.142±0.01	45.63±1.52
	2	164.00±3.90	2.174±0.02	40.78±1.63
	3	152.16±2.90	2.092±0.01	42.25±1.50
	4	146.09±2.50	2.084±0.01	44.89±1.47
0.20	1	150.69±2.75	2.136±0.01	44.73±1.50
	2	151.32±2.96	2.141±0.01	45.59±1.47
	3	149.20±3.30	2.113±0.02	44.85±1.63
	4	153.92±3.61	2.145±0.01	43.07±1.69
0.25	1	152.02±2.65	2.214±0.01	45.68±1.44

	2	153.56±3.19	2.764±0.02	42.60±1.85
	3	151.39±2.98	2.122±0.01	44.51±1.53
	4	162.47±5.88	2.967±0.02	41.24±1.54
0.30	1	164.08±4.01	3.150±0.03	40.03±1.65
	2	170.90±4.68	3.300±0.03	38.79±1.76
	3	166.89±2.77	2.577±0.02	39.43±1.57
	4	161.30±3.17	2.331±0.01	40.76±1.60
0.35	1	166.54±4.33	3.398±0.02	40.30±1.58
	2	172.34±6.90	4.006±0.03	35.78±2.05
	3	171.71±5.03	2.949±0.03	39.04±1.82
	4	168.14±5.07	3.403±0.02	40.44±1.62
0.40	1	184.36±4.32	4.179±0.02	35.32±1.83
	2	167.69±3.63	3.095±0.05	38.33±1.84
	3	192.05±6.73	4.167±0.02	35.28±1.75
	4	168.07±4.01	4.081±0.01	38.83±1.80
Added counter ion Cl-neutral model system				
0.40	1	163.22±3.12	3.795±0.02	38.68±1.59
	2	177.73±2.75	4.171±0.02	37.74±1.47
	3	172.21±2.36	4.047±0.02	39.29±1.56
	4	177.03±3.85	4.010±0.02	38.42±1.68

Disclaimer/Publisher's Note: The statements, opinions and data contained in all publications are solely those of the individual author(s) and contributor(s) and not of MDPI and/or the editor(s). MDPI and/or the editor(s) disclaim responsibility for any injury to people or property resulting from any ideas, methods, instructions or products referred to in the content.
